# Propranolol is efficacious against *Aspergillus* and *Fusarium* corneal isolates *in vitro* and in a murine model of *Aspergillus* keratitis

**DOI:** 10.1128/aac.01664-24

**Published:** 2025-05-15

**Authors:** Michael E. Zegans, Manali M. Kamath, Jane T. Jones, Ruina Bao, Brandon S. Ross, Cecilia Gutierrez-Perez, Emily M. Adams, Jorge D. Lightfoot, Galini Poimenidou, Chetan Pavuluri, Venkatesh Prajna, Robert A. Cramer, Kevin K. Fuller

**Affiliations:** 1 Department of Microbiology and Immunology, Geisel School of Medicine at Dartmouth 12285, Hanover, New Hampshire, USA; 2 Department of Surgery, Section of Ophthalmology, Dartmouth Hitchcock Medical Center 207077, Lebanon, New Hampshire, USA; 3 Deparment of Ophthalmology, University of Pittsburgh School of Medicine 12317 , Pittsburgh, Pennsylvania, USA; 4 Department of Ophthalmology, The University of Oklahoma Health Sciences Center 6186, Oklahoma City, Oklahoma, USA; 5 Department of Biology, Lawrence University 1279, Appleton, Wisconsin, USA; 6 Department of Cornea, Aravind Eye Hospital 29954, Madurai, India; 7 Department of Microbiology and Immunology, University of Oklahoma Health Sciences Center 6186, Oklahoma City, Oklahoma, USA; University of Iowa, Iowa City, Iowa, USA

**Keywords:** fungal keratitis, propranolol, *Fusarium*, *Aspergillus*, antifungal agents, keratitis

## Abstract

Fungal keratitis is a severe corneal infection most commonly caused by filamentous fungi. Even with prompt treatment with current antifungals, it often results in corneal perforation and blindness. In this report, we observe that the beta-adrenergic antagonist, propranolol, displays antifungal activity against *Aspergillus* and *Fusarium* corneal isolates *in vitr*o and strikingly blocks disease establishment in a murine model of *Aspergillus fumigatus* keratitis. These findings suggest that beta-blockers have potential as a novel FK treatment.

**CLINICAL TRIALS:**

This study is registered with ClinicalTrials.gov as NCT00997035 (MUTT Trial).

## INTRODUCTION

Fungal keratitis (FK) is a severe and often blinding infection commonly caused by filamentous fungi. Despite the introduction of newer antifungals, such as the azoles, the polyene natamycin remains the only Food and Drug Administration-approved drug for FK treatment. Indeed, the Mycotic Ulcer Treatment Trials (MUTT-I and II) found that voriconazole, a drug-of-choice for many systemic mold infections, is inferior to natamycin as a topical monotherapy and provides minimal benefit as an oral supplement to topical therapy alone (1). Both studies revealed a high rate of corneal perforation and/or transplantation even with natamycin treatment. Transplanted corneas in the MUTT-II were culture positive in 67% cases. These findings, in agreement with other studies, indicate that currently available antifungals often fail to achieve a microbiologic cure, and fungal persistence drives poor clinical outcomes. The development of better FK therapies is therefore a clinical imperative. We previously reported that the beta-adrenergic antagonist (a.k.a. beta-blocker) timolol is not intrinsically antifungal but synergizes with natamycin against filamentous fungi at concentrations comparable to those used to treat glaucoma (2). Interestingly, several studies have reported direct antifungal activity of another beta-blocker, propranolol, against *Candida albicans* and the plant pathogen *Magnaporthe oryzae* (3, 4). Here, we explore the activity of propranolol against FK-relevant molds and its potential use as an FK therapeutic *in vivo*.

**Fig 1:**
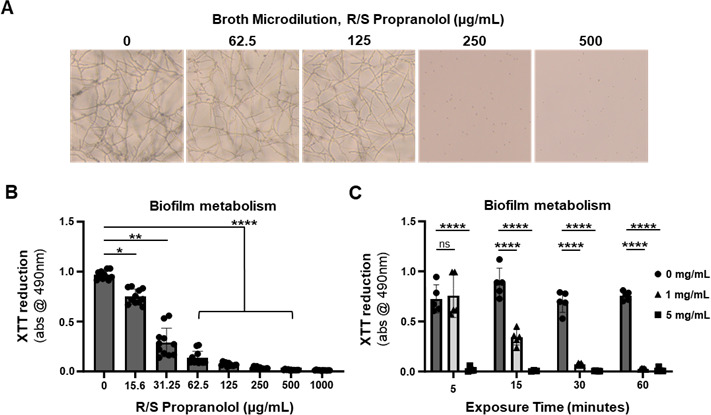
*In vitro* antifungal activity of R/S propranolol against *Aspergillus fumigatus*. (**A**) Conidia of *A. fumigatus* (Af293) were incubated in RPMI containing R/S propranolol at 35°C for 48 h. (**B**) Conidia of Af293 were incubated in glucose minimal media (GMM) for 24 h and subsequently washed and overlaid with RPMI media containing R/S propranolol at 35°C for 2 h. The metabolic activity of the biofilm was then analyzed by XTT assay, and groups were compared using ordinary one-way ANOVA, where asterisks reflect statistical significance relative to the untreated group based on Dunnett’s post hoc test for multiple comparisons. (**C**) Conidia of Af293 were incubated in GMM for 24 h and subsequently washed and overlaid with RPMI media containing R/S propranolol at 35°C for 2 h for the indicated times. The metabolic activity of the biofilm was then analyzed by XTT assay, and groups at each time point were compared using two-way ANOVA. Asterisks reflect statistical significance relative to the untreated group at each time point based on Dunnett’s post-hoc test for multiple comparisons: (ns) not significant; (****) *P* < 0.0001. For panels B and C: (ns) not significant; (*) *P* < 0.05; (****) *P* < 0.0001.

We began by screening the activity of R-, S-, and R/S-propranolol (Sigma-Aldrich) against six *Aspergillus fumigatus* strains, a common FK agent, in a standard broth microdilution assay. Briefly, conidia were harvested from glucose minimal media (GMM) plates and inoculated into RPMI-1640 media at a final density of 5.0 × 10^4^/mL in 200 µL (2). Following 48 h incubation at 37°C (atmospheric conditions), both enantiomers and the racemic mixture of propranolol inhibited germination of each strain at 125–250 µg/mL (Table 1 and Fig. 1A). To determine if this antifungal activity extended to the hyphal (tissue invasive) form of the fungus, biofilms of strain Af293 were pre-formed in 96-well plates overnight in GMM broth, washed and overlaid with R/S-propranolol containing RPMI-media, and incubated for 2 h before measuring the metabolic activity of the biomass using the 2,3-bis-(2-methoxy-4-nitro-5-sulphenyl)-(2H)-tetrazolium-5-carboxanilide (XTT) reduction assay. Interestingly, though a complete loss in metabolic activity was noted at concentrations that corresponded to the above-described conidial MICs (125–250 µg/mL), statistically relevant decreases in XTT reduction were noted as low as 16 µg/mL (Fig. 1B). Taken together, these results indicate that propranolol has antifungal activity below its MIC and is active against both *Aspergillus* conidia and biofilms.

**TABLE 1:** Microdilution broth assay of propranolol against *A. fumigatus* and *Fusarium* isolates^*a*^

Species (isolate)	R-propranolol MIC (µg/mL)	S-propranolol MIC (µg/mL)	R/S-propranolol MIC (µg/mL)
*Aspergillus fumigatus*			
Af293	250	125	250
A1163	125	125	125
IFM59356-1	125	125	125
IFM59356-3	250	250	250
Af110-5.3	250	250	125
Af100-10.1	250	250	250
MIC range	125–250	125–250	125–250
			
*Fusarium* (FSSC)			
MUTT 1121U	52	52	31
MUTT 1146N	52	52	63
MUTT 1584Q	52	63	52
MUTT 1587W	47	42	42
MUTT 1736Z	31	42	42
MUTT 1753Z	31	42	31
MUTT 2725Z	42	63	52
MUTT 2226R	47	52	52
UCSF 06-0110	42	52	52
UCSF 06-0111	26	52	42
UCSF 06-0133	26	52	31
UCSF 06-0330	26	52	42
UCSF 06-0487	52	63	31
DUMC 132.02	31	73	73
*Fusarium* (FOSC)			
UCSF 06–0197	42	52	52
UCSF 06–0342	42	63	52
*Fusarium* (FDSC)			
MUTT 1090Z	83	104	83
*Fusarium* (FFSC)			
MUTT-1170W	27	42	42
MIC Range	27–83	42–104	31–83

^*a*^ Conidia of the indicated strains were incubated in the presence of R-, S-, or R/S- propranolol in RPMI-1640 media. *A. fumigatus* strains were incubated at 37°C for 48 h, and *Fusarium* strains were incubated at 30°C for 72 h before recording the MIC. Values reflect the average MIC from two independent experiments. The 10 MUTT isolates selected from the MUTT II collection were selected randomly for this study using random.org. The UCSF isolates are from the University of California at San-Francisco-Proctor Foundation and DUMC 132.02 is from Duke University Medical Center (courtesy of Dr. Wiley Schell). MIC, minimum inhibitory concentration; FSSC, *Fusarium solani* species complex; FOSC, *Fusarium oxysporum* species complex; FFSC, *Fusarium fujikuroi* species complex.

**Fig 2:**
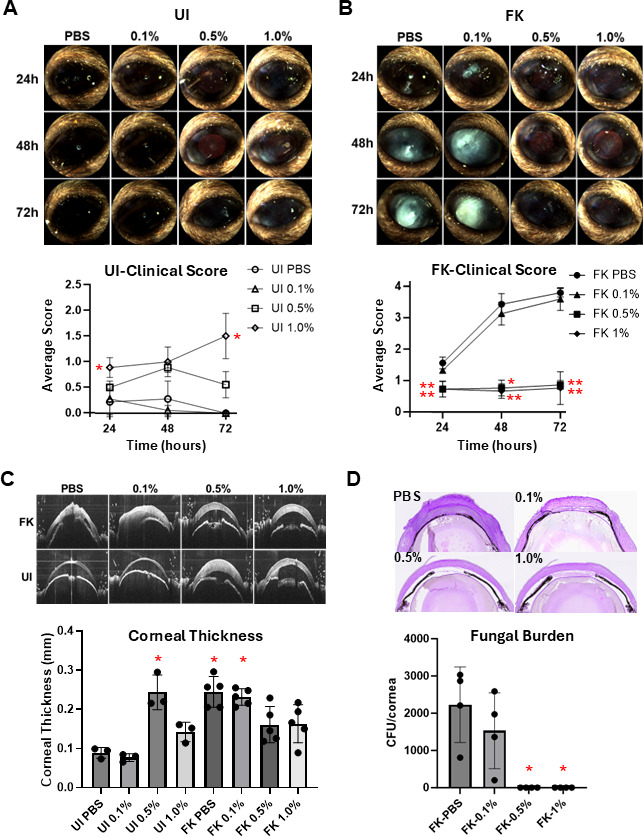
Topical propranolol reduces fungal growth and disease burden in a murine model of *Aspergillus fumigatus* fungal keratitis. (A–B) Representative slit-lamp images of uninfected (UI) or *A. fumigatus*-infected (FK) corneas treated with the indicated concentration of topical R/S-propranolol or vehicle (PBS). All treated animals also received daily intraperitoneal injections of 2 mg/kg propranolol. Mean clinical disease scores are plotted (±SD) (*n* = 5/treatment group). Groups were compared at each time point by Kruskal-Wallis test, and asterisks reflect statistical significance relative to either the UI-PBS (panel A) or FK-PBS (panel B) groups based on Dunn’s post-hoc test for multiple comparisons. (**C**) OCT scans of UI and FK corneas (the same as panels A and B), taken at 72 h p.i. Mean corneal thickness measurements based on the OCT images are plotted (±SD). Groups were compared by Kruskal-Wallis test, and asterisks reflect statistical significance relative to the UI-PBS group based on Dunn’s post-hoc test for multiple comparisons. (**D**) At 72 h p.i., eyes were either resected for PASH staining (1/group) or homogenized for CFU analysis (4/group). Mean CFUs (±SD) are plotted. Groups were compared by the Kruskal-Wallis test, and asterisks reflect statistical significance relative to the FK-PBS group based on Dunn’s post-hoc test for multiple comparisons. For all panels: (*) *P* < 0.05; (**) *P* < 0.01.

To assess the antifungal activity of propranolol on the ocular surface, we utilized an established corneal abrasion (Algerbrush) model of FK using immunosuppressed C57BL/6J mice and *A. fumigatus* strain Af293 (5–8). On the day preceding fungal inoculation, animals began a once-daily treatment with 2 mg/kg R/S-propranolol by intraperitoneal (i.p.) injection, which recapitulates a prior report in which such treatments reduced kidney fungal burden in a murine model of systemic candidiasis (9). Unique to our study, animals further received a single topical treatment of 1 mg/mL (0.1%), 5 mg/mL (0.5%), or 10 mg/mL (1%) propranolol diluted in PBS on day 0, several hours after fungal inoculation, by anesthetizing them with isoflurane and applying a 5 µL drop to the ocular surface for 5 min. On days + 1 and +2, animals received three topical treatments, 3 h apart, at the same concentration. Groups of five corneas (corresponding to five animals) were used for each topical treatment concentration in addition to a sham-treated group that received both systemic and topical PBS on the same schedule. The contralateral eyes were used as sham-inoculated (uninfected, UI) controls and received the same treatment as the infected (FK) eye. Corneal pathology was tracked longitudinally by slit-lamp (Micron IV) for clinical disease scoring and by optical coherence tomography (OCT) to quantify corneal thickness, as previously described (5–8). Eyes were harvested at 72 h to quantify fungal burden, based on colony forming units (CFU), or for histological endpoints. As shown in Fig. 2, FK corneas that were treated with either PBS or 0.1% topical propranolol developed a progressive corneal opacification that was associated with increased tissue thickness (edema) (Fig. 2C), fungal load, and inflammation at 72 h post-inoculation (p.i.). By contrast, eyes treated with 0.5% or 1.0% topical propranolol remained largely indistinguishable from uninfected control eyes, and this corresponded to an absence of viable fungus and minimal inflammation at end point. Congruent with our *in vivo* data, when Af293 biofilms were incubated with 0.5% propranolol *in vitro*, a complete loss in fungal metabolism (XTT reduction) was observed in as early as 5 min; by contrast, it took 30 min of exposure to 0.1% propranolol to achieve the same effect (Fig. 1C). These results indicate that topical propranolol can exert antifungal effects on the ocular surface of mice in a dose-dependent manner.

Interestingly, uninfected corneas that received 0.5 and 1% topical propranolol, but not the 0.1% concentration, displayed signs of corneal edema based on OCT, despite appearing largely healthy based on slit-lamp scores (Fig. 2A and C). While topical natamycin has also been reported to cause ocular surface disease including edema (10), it is nevertheless intriguing to speculate that propranolol at the higher concentrations may be altering corneal physiology. Topical 1% propranolol was investigated in the past for use in glaucoma (11). More recently, a trial was conducted with topical 0.2% propranolol for the treatment of retinopathy of prematurity in infants (12). While we did not find reports of propranolol inducing corneal edema, further studies will be required to better understand these findings.

To gain additional insight into the potential scope of propranolol’s clinical utility in FK, we conducted susceptibility testing against 18 *Fusarium* corneal isolates obtained from India (the MUTT1 and 2 studies) as well as the United States (1, 2). Microconidia were collected from potato dextrose broth cultures, set up in a broth microdilution assay as described above for *A. fumigatus*, and incubated for 72 h incubation at 30°C. Remarkably, the three propranolol formulations inhibited all tested *Fusarium* isolates and did so at a lower concentration range (27–104 µg/mL) than was observed for *A. fumigatus* (Table 1).

In summary, propranolol has antifungal activity against the FK-relevant molds, *A. fumigatus* and *Fusarium spp*. Although the mechanism of action of the drug against these fungi remains an open area of investigation, the fact that the R- and S- enantiomers display comparable efficacy, as well as the apparent lack of beta-adrenergic receptor orthologs in the fungi, suggests that propranolol’s activity against fungi is distinct from its cardiovascular effects in humans (13). Prior work in both *Candida* and *Magnaporthe* observed propranolol-mediated inhibition of PLD1 and MoPah1 enzymes, respectively, both of which convert phosphatidic acid to diacylglycerol and are therefore involved in lipid metabolism and membrane homeostasis (3, 4). Similarly, propranolol (racemic and the R and S enantiomers) has been observed to block this pathway in humans via inhibition of phosphatidate phosphohydrolase (14). We are pursuing this and other possible mechanisms in *Aspergillus* and *Fusarium*, and such efforts may reveal novel proteins/pathways important for FK pathogenesis. Mechanism aside, the feasibility of developing propranolol as an FK therapeutic is supported by the fact that it has been safely used on the human ocular surface at concentrations similar to those tested here (11, 12). Finally, the evaluation of propranolol in a *Fusarium* FK model, its synergism with natamycin and other antifungals, and the effects of the drug on corneal biology/immunology will be important next steps in evaluating the therapeutic potential of propranolol for FK.
